# Supplementary material for: Divergent architecture of the heterotrimeric NatC complex explains N-terminal acetylation of cognate substrates
Source: Nat Commun. 2020 Nov 2;11:5506. doi: 10.1038/s41467-020-19321-8 (PMC7608589; doi:10.1038/s41467-020-19321-8)
Supplement: Supplementary file 1 — Supplementary Information [file 41467_2020_19321_MOESM1_ESM.pdf]

Supplementary Information

Divergent architecture of the heterotrimeric NatC complex explains N-terminal acetylation of cognate substrates

Grunwald et al.

Content

Supplementary Table 1: Primers sequences for vector construction and site-directed mutagenesis

Supplementary Fig. 1: Sequence alignment of Naa30 and Naa38 orthologs

Supplementary Fig. 2: Sequence alignment of Naa35 orthologs

Supplementary Fig. 3: MALDI-MS analysis of Naa38 and purification of NatC

Supplementary Fig. 4: Structural comparison of NatC subunits Naa30 and Naa38

Supplementary Fig. 5: NatC subunit interfaces

Supplementary Fig. 6: Michaelis-Menten kinetics of NatC for various substrate peptides

Supplementary Fig. 7: Michaelis-Menten kinetics for NatC mutants

Supplementary Fig. 8: Electron density maps for the active site of NatC

Supplementary Fig. 9: Comparisons of the peptide-binding surfaces in NAT orthologs

Supplementary Fig. 10: Peptide ligand-induced conformational changes

Supplementary Fig. 11: Replicates of the NatC/ribosome co-sedimentation assay

Supplementary Fig. 12: Replicates of the yeast dilution spot assay

Supplementary Fig. 13: Models of NatC on the ribosomal surface

Supplementary References

Supplementary Table 1 Primers sequences for vector construction and site-directed mutagenesis

**Primer name** (cloning strategy)	**Sequence (5' to 3')**
**For construction of NatC bacterial expression vectors**	
RBS-Naa30 Fw (PvuI)	GGCCGATCGAAGGAGATATACCATGGAAATAGTGTACAAGCCATTG
Naa30ΔC17 Rv (XhoI)	GGCCTCGAGTCATTAAGTCAACGGCAATATCAGTTTAAAG
HRV3C-Naa35 Fw (BamHI)	GGCGGATCCGCTGGAAGTTCTGTTCCAGGGGCCCATGGAAGTAGACAGTATATTAG
HRV3C-Naa35ΔN17 Fw (BamHI)	GGCGGATCCGCTGGAAGTTCTGTTCCAGGGGCCCCAATTGGTTGACGTCACAAG
HRV3C-Naa35ΔN44 Fw (BamHI)	GGCGGATCCGCTGGAAGTTCTGTTCCAGGGGCCCGATTTGTTCGAGGGTACAC
HRV3C-FLAG-Naa35 Fw (BamHI)	GGCGGATCCGCTGGAAGTTCTGTTCCAGGGGCCCGATTATAAAGATGATGATGATAAAATGGAAGTAGACAGTATATTAG
Naa35 Rv (NotI)	GGCGCGGCCGCTTATTTATAGCGGTCTTGC
Naa38 Fw (NdeI)	GGCCATATGGACATCTTGAAACTGTC
Naa38 Rv (MfeI)	GGCCAATTGCTAAACAATATTAGCCATCAATTC
Naa38ΔC11 Fw (site-directed mutagenesis)	CTGCAGGAGCTTACTTAGCAATTGGATATC
Naa38ΔC11 Rv (site-directed mutagenesis)	GATATCCAATTGCTAAGTAAGCTCCTGCAG
**For construction of FLAG-Naa35 yeast expression vectors**	
pRS416_Naa35 Fw (Gibson[1])	TCGAATTCCTGCAGCCCGGGGCCAGCAAATACTATGTTTCACTGC
pRS416_Naa35 Rv (Gibson)	GCGGCCGCTCTAGAACTAGTGCGGGATTTTCCCTTCGGTTAC
FLAG-Naa35 Fw (Gibson)	ATGGATTATAAAGATGATGATGATAAAATGGAAGTAGACAGTATATTAGG
FLAG-Naa35 Rv (Gibson)	TTTATCATCATCATCTTTATAATCCATTCTCGGAAACGTACCAGC
**For generation of NatC-Naa30 mutations by site-directed mutagenesis**	
L27A_Fw	GTATTAAAAAACTAATAGATGCGGATGCATCAGAGCCGTACTCGATATAC
L27A_Rv	GTATATCGAGTACGGCTCTGATGCATCCGCATCTATTAGTTTTTTAATAC
S28A_Fw	CTAATAGATGCGGATCTAGCAGAGCCGTACTCGATATAC
S28A_Rv	GTATATCGAGTACGGCTCTGCTAGATCCGCATCTATTAG
E29A_Fw	GGATCTATCAGCGCCGTACTCG
E29A_Rv	CGAGTACGGCGCTGATAGATCC
E29Q_Fw	GGATCTATCACAGCCGTACTCG
E29Q_Rv	CGAGTACGGCTGTGATAGATCC
Y31F_Fw	GGATCTATCAGAGCCGTTCTCGATATACGTATATAGGTATTTC
Y31F_Rv	GAAATACCTATATACGTATATCGAGAACGGCTCTGATAGATCC
Y80A_Fw	CGTGAGACTGAGAGGAGCTATAGGAATGCTAGCCGTAG
Y80A_Rv	CTACGGCTAGCATTCCTATAGCTCCTCTCAGTCTCACG
Y80F_Fw	GACTGAGAGGATTTATAGGAATGC
Y80F_Rv	GCATTCCTATAAATCCTCTCAGTC
E118A_Fw	GATCATGTTAGCAACAGAGGTGG
E118A_Rv	CCACCTCTGTTGCTAACATGATC
E118Q_Fw	GATCATGTTACAAACAGAGGTGG
E118Q_Rv	CCACCTCTGTTTGTAACATGATC
E120A_Fw	GTTAGAAACAGCGGTGGAAAATTC
E120A_Rv	GAATTTTCCACCGCTGTTTCTAAC
E120Q_Fw	GTTAGAAACACAGGTGGAAAATTC
E120Q_Rv	GAATTTTCCACCTGTGTTTCTAAC
Y130A_Fw	GCGGCTCTAAACTTAGCTGAAGGAATGGGTTTCATC
Y130A_Rv	GATGAAACCCATTCCTTCAGCTAAGTTTAGAGCCGC
Y130F_Fw	GCGGCTCTAAACTTATTTGAAGGAATGGGTTTCATC
Y130F_Rv	GATGAAACCCATTCCTTCAAATAAGTTTAGAGCCGC
Y145F_Fw	GAAACGGATGTTCCGCTACTTCTTGAATGAAGGGGACG
Y145F_Rv	CGTCCCCTTCATTCAAGAAGTAGCGGAACATCCGTTTC

**For generation of NatC-Naa35 mutations by site-directed mutagenesis**	
F47A_Fw	GATCCCAGGTTTGATTTGGCCGAGGGTACACATTCTTTAG
F47A_Rv	CTAAAGAATGTGTACCCTCGGCCAAATCAAACCTGGGATC
K59A_Fw	CTTTAGAGGTCAACAACTCCGCATTAGACTCCAGTCTGATAG
K59A_Rv	CTATCAGACTGGAGTCTAATGCGGAGTTGTTGACCTCTAAAG
Naa35-EPR1[a] Fw	GCTGCAAACGTGGCACTGGCAGGATATATAGGAATGCTAGC
Naa35-EPR1 Rv	TGCCAGTGCCACGTTTGCAGCCGGGTCCATCTTACACACTAT
Naa35-EPR2-Tip1[b] Fw	GAACGCAGCACTTGCAGCATTGAAAGCTGGTGAAAAG
Naa35-EPR2-Tip1 Rv	GCTGCAAGTGCTGCGTTCATGGAATGAATGGC
Naa35-EPR2-Tip2[c] Fw	GACCAATTGGCGCTAAAGTACGCGTTTGCTATGGATAATGAAATG
Naa35-EPR2-Tip2 Rv	CTTTAGCGCCAATTGGTCGGCCTTTTCACCAGCTTTCAAT
Naa35-EPR3[d]-a Fw	CTTTATCGAAACTGAAGTGGCCAATGTCGTTAGTTCTATTG
Naa35-EPR3-a Rv	CAATAGAACTAACGACATTGGCCACTTCAGTTTCGATAAAG
Naa35-EPR3-b Fw	GCATTATCAGTCAACTCAGCAGTTATCGTCAACACTTTG
Naa35-EPR3-b_Rv	CTGAGTTGACTGATAATGCTGCAGAAGTATGTTTTAGTTTACAATAG
Naa35-EPR4[e] Fw	GCGAACATTGCAAACGCGGACTCCCACGAATACAAG
Naa35-EPR4 Rv	CGCGTTTGCAATGTTCGCTTTCAAAGTGTTGACGATAAC

RBS, ribosome-binding site HRV3C, Human Rhinovirus (HRV) 3C protease cleavage site (LEVLFQGP); FLAG, FLAG-epitope (DYKDDDDK); EPR, Electropositive region

[a]The Naa35-EPR1 primer pair generates Naa30 point mutations H72A, R73A, R76A and R78A.

[b]The Naa35-EPR2-Tip1 primer pair generates Naa35 point mutations K500A, K501A, K503A and K504A.

[c]The Naa35-EPR2-Tip2 primer pair generates Naa35 point mutations K511A, R515A, and R519A.

[d]The two Naa35-EPR3 primer pairs were used successively to generates Naa35 point mutations R636A, K681A, and K688A.

[e]The Naa34-EPR4 primer pair generates Naa35 point mutations K696A, K699A and K701A.

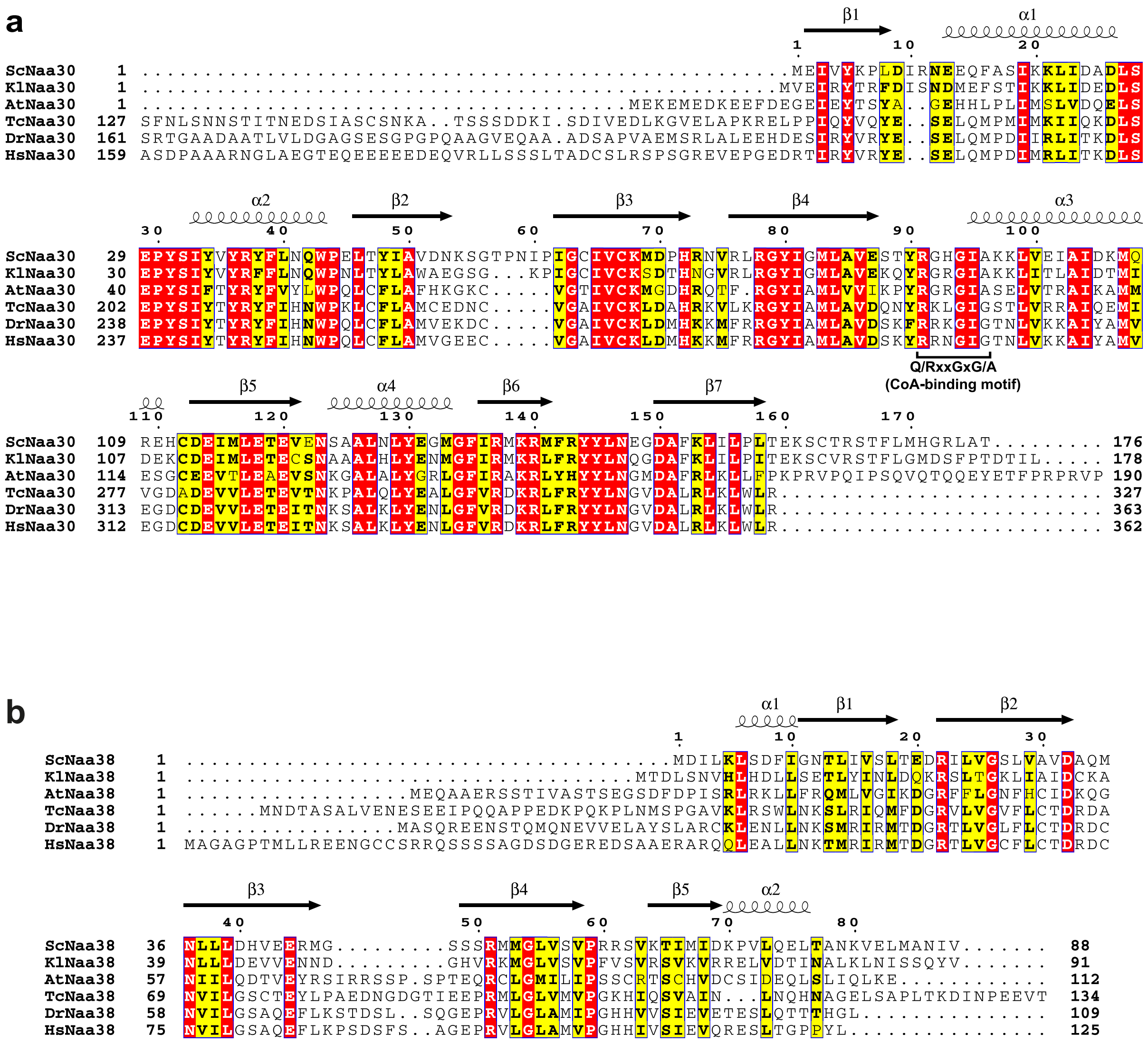

Supplementary Fig. 1 Sequence alignment of Naa30 and Naa38 orthologs. a, **b** Completely conserved residues are highlighted with red boxes, highly conserved with yellow boxes. Secondary structures assignments are shown on top. The following sequences of Naa30 and Naa38 were aligned: Sc, *Saccharomyces cerevisiae* (accession numbers NP_015376.1 and NP_009948.1); Kl, *Kluyveromyces lactis* (XP_452068.1 and XP_451488.1), At, *Arabidopsis thaliana* (NP_181348.1 and NP_680719.1); *Tc, Tribolium castaneum* (XP_975323.1 and XP_008192183.1); Dr, *Danio rerio* (NP_001129721.2 and NP_001153763.1) and Hs, *Homo sapiens* (NP_001011713.2 and NP_001307854.1).

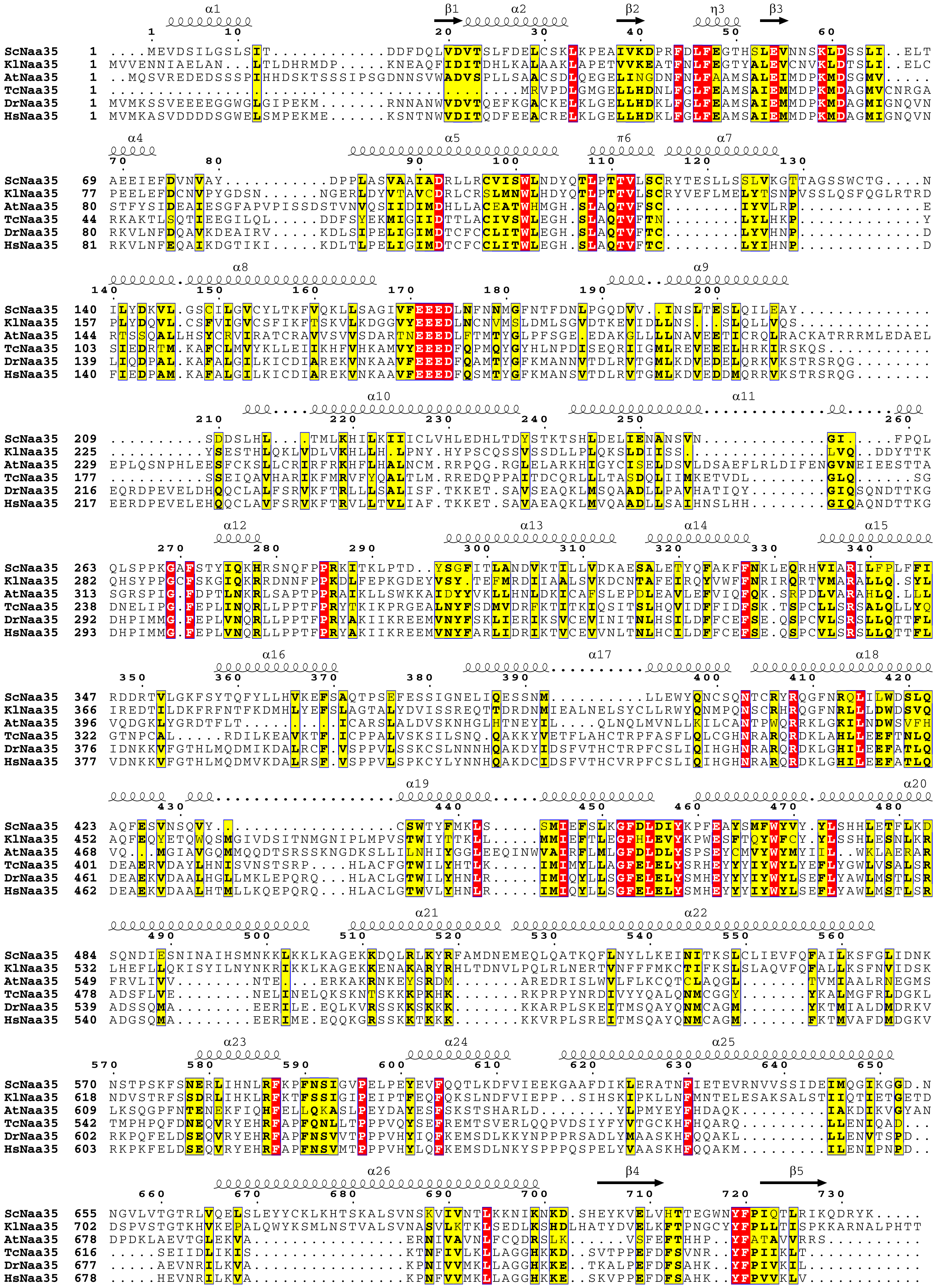

Supplementary Fig. 2 Sequence alignment of Naa35 orthologs. Completely conserved residues are highlighted with red boxes, highly conserved residues with yellow boxes. Secondary structures assignments of ScNaa35 are indicated on top. The following Naa35 sequences were aligned: Sc, *Saccharomyces cerevisiae* (accession number NP_010861.3); Kl, *Kluyveromyces lactis* (XP_451085.1); At, *Arabidopsis thaliana* (NP_178872.2)*; Tc, Tribolium castaneum* (XP_008198377.1); Dr, *Danio rerio* (NP_955844.1); and Hs, *Homo sapiens* (NP_078911.3).

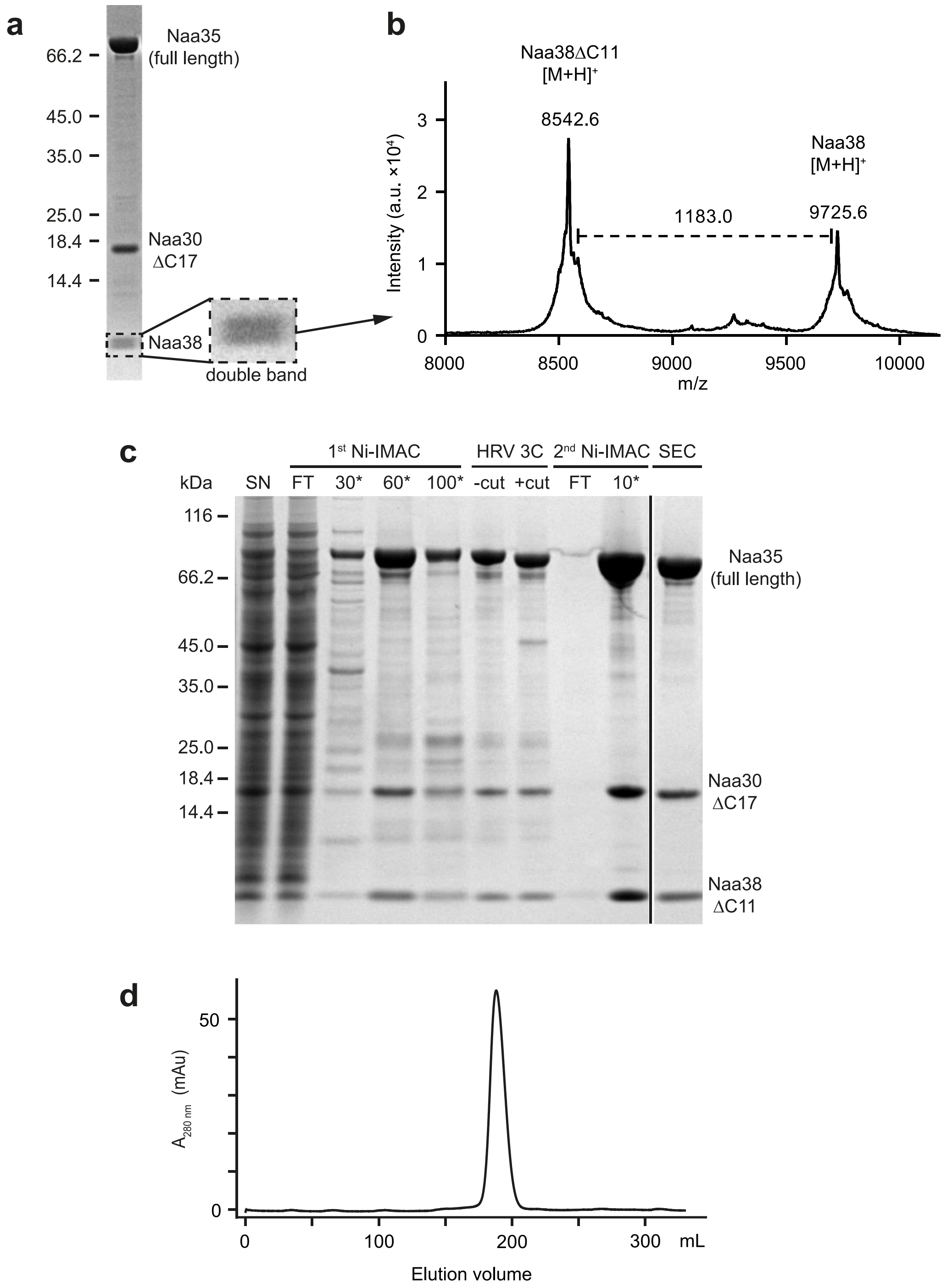

Supplementary Fig. 3 MALDI-MS analysis of Naa38 and purification of NatC. a During an initial purification of a NatC complex containing subunits Naa30ΔC17 (residues 1–159), full-length Naa35 (residues 1–733) and full-length Naa38 (residues 1–88), a partial proteolytic degradation of the small subunit Naa38 was observed. The purification of this NatC construct was performed only once. **b** MALDI-MS analysis of the protein purification shown in subfigure a. The right peak corresponds to full-length Naa38 (theoretical MW = 9724.6 Da) and the left peak was attributed to a partial proteolytic degradation product of Naa38, which lacks eleven C-terminal residues (Naa38ΔC11, theoretical MW = 8541.1 Da). **c** Samples collected from a purification of NatC WT containing subunits Naa30ΔC17, Naa38ΔC11 and full-length Naa35. The latter is preceded by an N-terminal 6×His-tag, followed by a human rhinovirus 3C cleavage site. Purifications of NatC WT were repeated three times with identical results. Ni-IMAC, Ni^{2+}-immobilized metal ion affinity chromatography; SN, supernatant; FT, flow-through; *, Imidazole concentrations during wash and elution steps; HRV 3C, human rhinovirus 3C protease cleavage with fractions before (-cut) and after (+cut) digest; SEC, size exclusion chromatography. The juxtaposed SEC lane is from the same gel. **d** Size exclusion chromatography profile for the NatC complex, run on a S200, 26/600 column. Uncropped images for panels a and c are provided in the Source Data file.

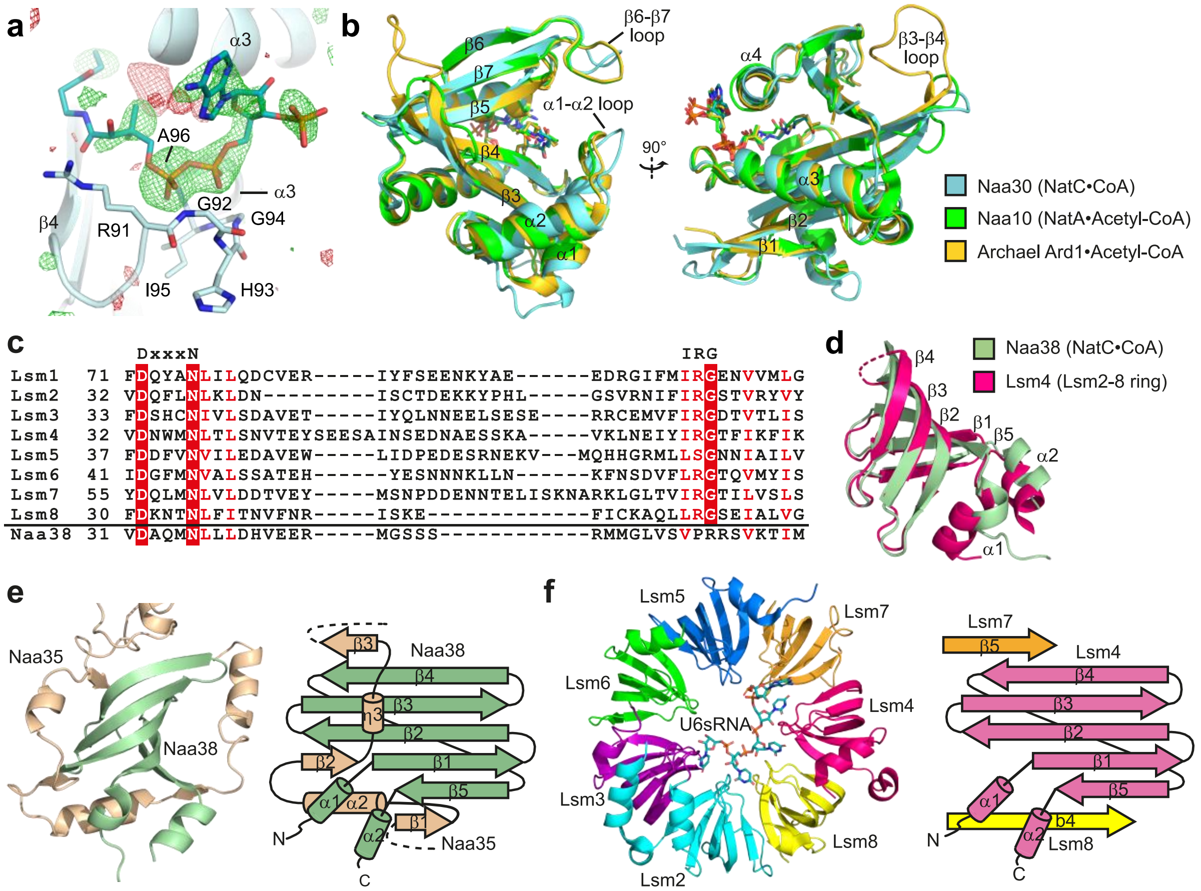

Supplementary Fig. 4 Structural comparison of NatC subunits Naa30 and Naa38. a CoA (teal stick format) bound to the Naa30 subunit of the NatC•CoA structure. A simulated annealing omit map contoured to 3.0 σ shows positive (green) and negative (red) difference electron density. Residues within the conserved CoA-binding motif RxxGxG/A are shown as sticks. **b** Superposition of Naa30 with its closest structural homologs. The RMSD between Naa30 and the archaeal Ard1 NAT ortholog from *Sulfolobus solfataricus* (4LX9) is 1.6 Å (over 149 Cα atoms) and between Naa30 and the Naa10 subunit of the *Schizosaccharomyces pombe* NatA complex (4KVO) complex 1.7 Å (over 150 Cα atoms). **c** Sequence alignment of the *S. cerevisiae* Naa38 and Lsm1-8 proteins showing the region between the conserved DxxxN and IRG motifs. Completely conserved residues are highlighted with red boxes, highly conserved residues are colored in red. **d** Superposition of Naa38 with its closest structural homolog Lsm4 (RMSD of 1.8 Å, over 69 Cα atoms) of the heptameric Lsm2-8 ring (4M7A). **e**, **f** Comparison of the β-sheet topology of the Naa35-Naa38 β-sheet (**e**) with the β-sheet topology of Lsm4 in contact with its neighboring Lsm units (Lsm7 and Lsm8) within the Lsm2-8 ring (**f**).

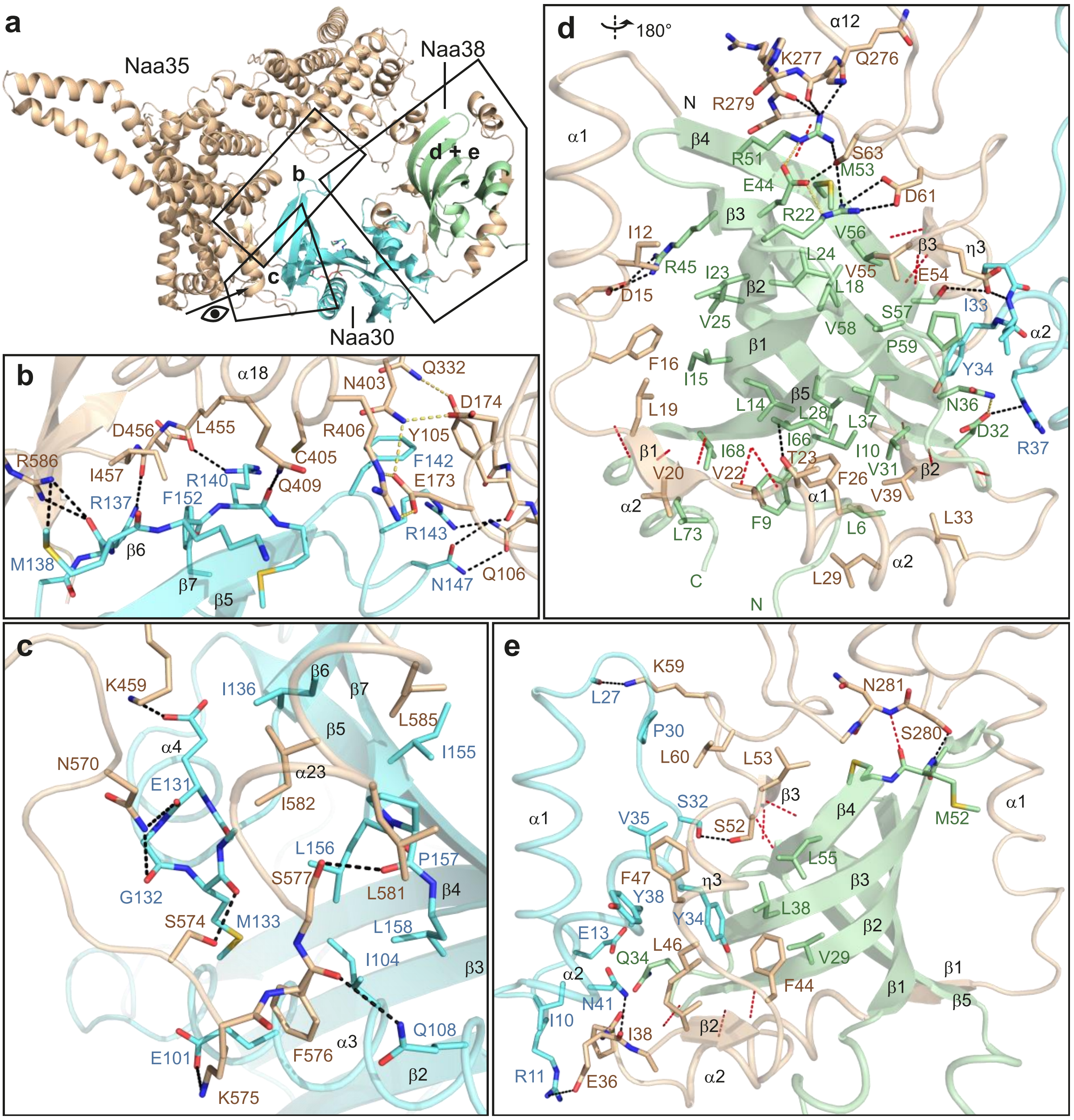

Supplementary Fig. 5 NatC subunit interfaces. a Cartoon representation of the NatC•CoA structure. **b**, **c** Magnified views of the Naa30-Naa35 interface, which is dominated by hydrogen-bonding interactions (dashed lines, colored in black) and involves the C-terminal half (residues 101–158) of the Naa30 subunit. Key interface residues are shown in stick format. **d**, **e** Magnified views of the interfaces between the Naa38 subunit, the Naa35 N-terminus (residues 1–64), a short stretch around Naa35-helix α12 (residues 276–282) and the N-terminal half of the Naa30 subunit (residues 10–41). The interface between Naa38 and Naa35 is dominated by hydrophobic side chain interactions. Additionally, the β-sheet of Naa38 is extended by three short β-strands (β1–β3) from the Naa35 N-terminus (backbone-backbone interactions are colored in red).

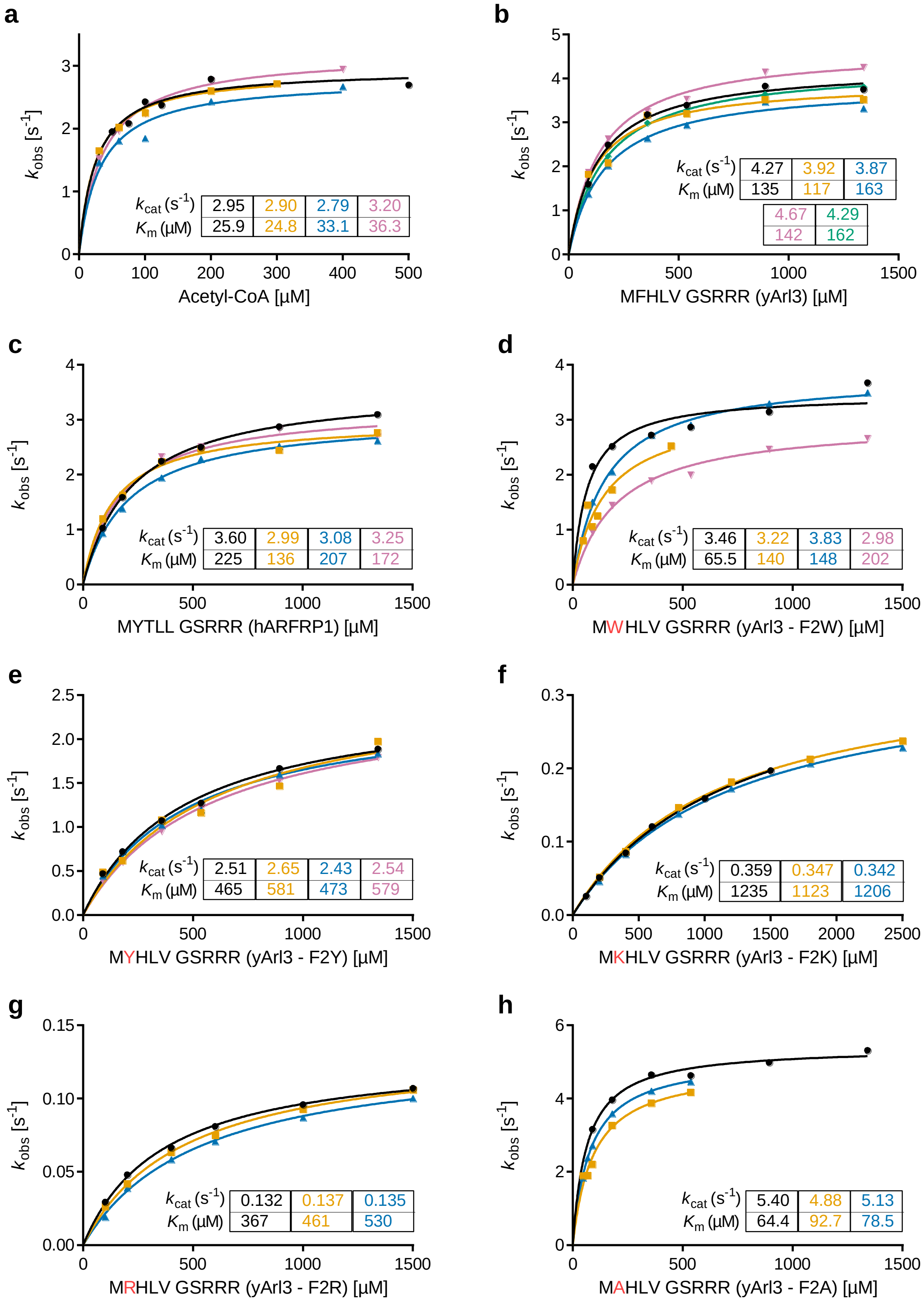
a
kobs [s-1]
Acetyl-CoA [µM]
kcat (s-1) 2.95 2.90 2.79 3.20
Km (µM) 25.9 24.8 33.1 36.3
b
MFHLV GSRRR (yArl3) [µM]
kcat (s-1) 4.27 3.92 3.87
Km (µM) 135 117 163
4.67 4.29
142 162
c
MYTLL GSRRR (hARFRP1) [µM]
kcat (s-1) 3.60 2.99 3.08 3.25
Km (µM) 225 136 207 172
d
MWHLV GSRRR (yArl3 - F2W) [µM]
kcat (s-1) 3.46 3.22 3.83 2.98
Km (µM) 65.5 140 148 202
e
MYHLV GSRRR (yArl3 - F2Y) [µM]
kcat (s-1) 2.51 2.65 2.43 2.54
Km (µM) 465 581 473 579
f
MKHLV GSRRR (yArl3 - F2K) [µM]
kcat (s-1) 0.359 0.347 0.342
Km (µM) 1235 1123 1206
g
MRHLV GSRRR (yArl3 - F2R) [µM]
kcat (s-1) 0.132 0.137 0.135
Km (µM) 367 461 530
h
MAHLV GSRRR (yArl3 - F2A) [µM]
kcat (s-1) 5.40 4.88 5.13
Km (µM) 64.4 92.7 78.5

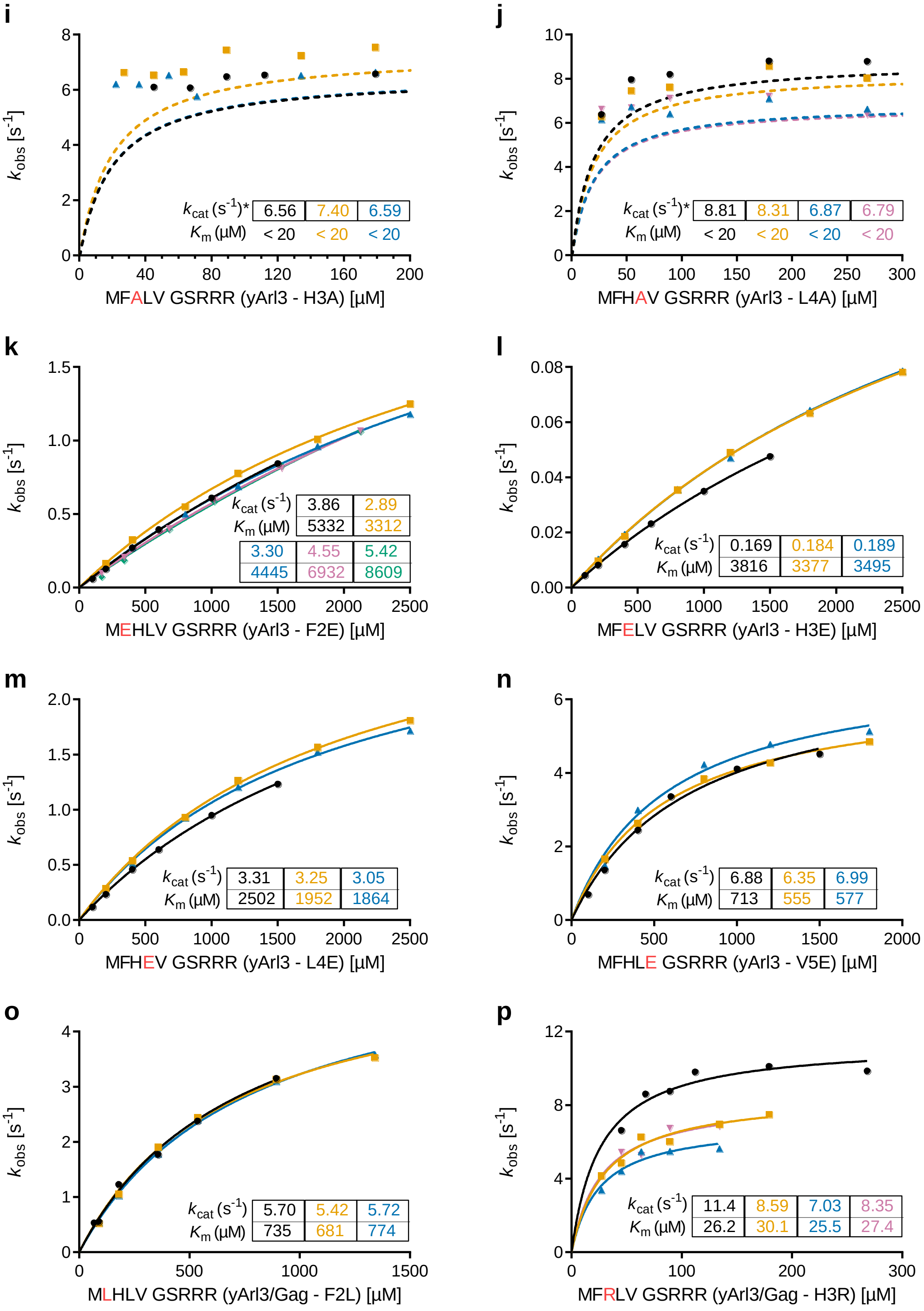
i
k_{obs} [s^{-1}]
k_{cat} (s^{-1})* 6.56 7.40 6.59
K_m (µM) < 20 < 20 < 20
MFALV GSRRR (yArl3 - H3A) [µM]
j
k_{obs} [s^{-1}]
k_{cat} (s^{-1})* 8.81 8.31 6.87 6.79
K_m (µM) < 20 < 20 < 20 < 20
MFHAV GSRRR (yArl3 - L4A) [µM]
k
k_{obs} [s^{-1}]
k_{cat} (s^{-1}) 3.86 2.89
K_m (µM) 5332 3312
3.30 4.55 5.42
4445 6932 8609
MEHLV GSRRR (yArl3 - F2E) [µM]
l
k_{obs} [s^{-1}]
k_{cat} (s^{-1}) 0.169 0.184 0.189
K_m (µM) 3816 3377 3495
MFELV GSRRR (yArl3 - H3E) [µM]
m
k_{obs} [s^{-1}]
k_{cat} (s^{-1}) 3.31 3.25 3.05
K_m (µM) 2502 1952 1864
MFHEV GSRRR (yArl3 - L4E) [µM]
n
k_{obs} [s^{-1}]
k_{cat} (s^{-1}) 6.88 6.35 6.99
K_m (µM) 713 555 577
MFHLE GSRRR (yArl3 - V5E) [µM]
o
k_{obs} [s^{-1}]
k_{cat} (s^{-1}) 5.70 5.42 5.72
K_m (µM) 735 681 774
MLHLV GSRRR (yArl3/Gag - F2L) [µM]
p
k_{obs} [s^{-1}]
k_{cat} (s^{-1}) 11.4 8.59 7.03 8.35
K_m (µM) 26.2 30.1 25.5 27.4
MFRLV GSRRR (yArl3/Gag - H3R) [µM]

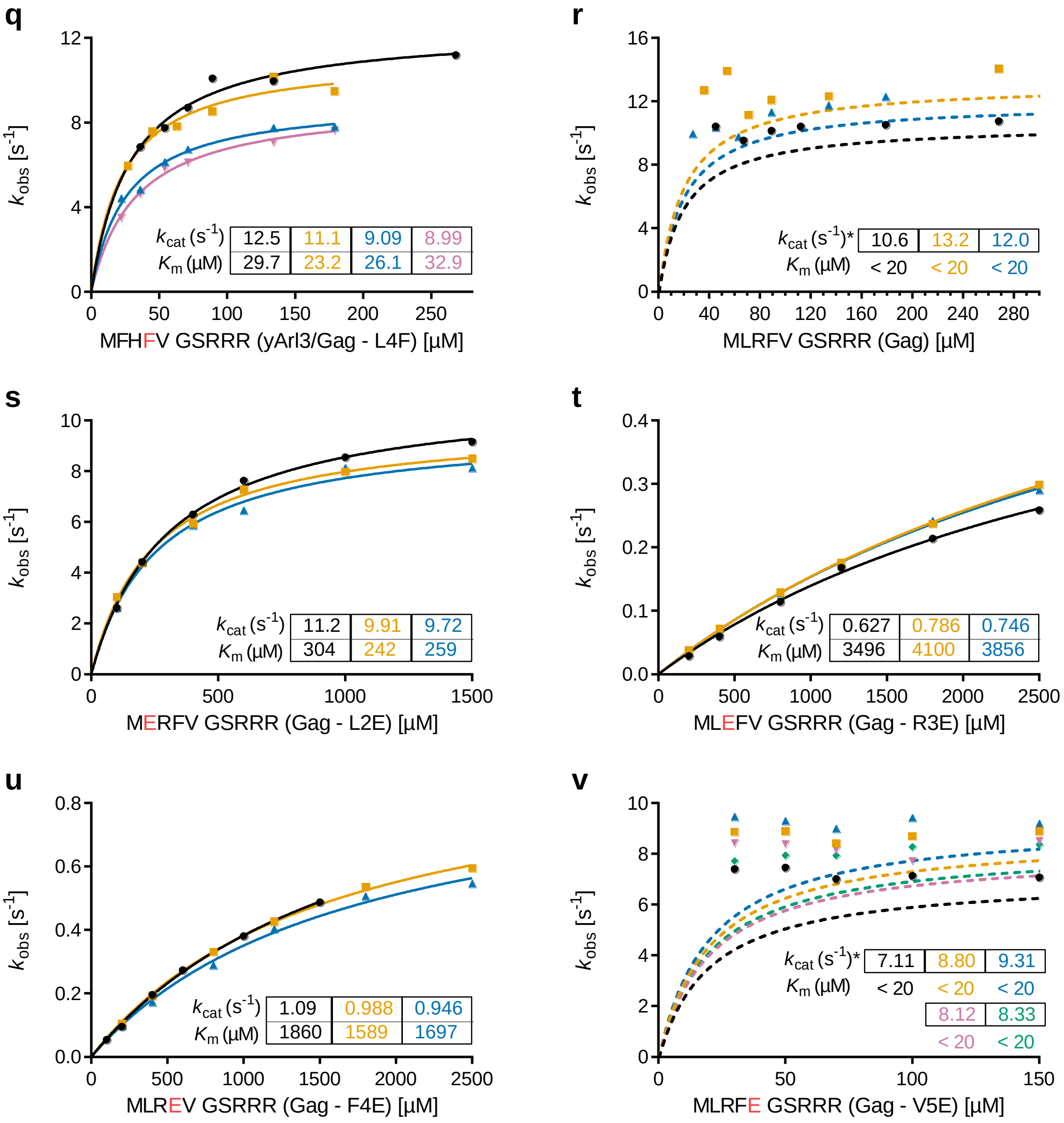

Supplementary Fig. 6 Michaelis-Menten kinetics of NatC for various substrate peptides. All reactions were performed with NatC WT (Naa30ΔC17, full-length Naa35, Naa38ΔC11). k_{obs} values represent initial reaction velocities (v_0) divided by the NatC concentration. Each graph (colored in black, orange, blue, purple and green) represents an independent experiment. Catalytic parameters (K_m, k_{cat}) for each replicate are shown below the plots in the corresponding color. **a** Kinetics for acetyl-CoA (variable substrate) with the yArl3 peptide (MFHLVGSRRR) at a concentration of 1340 µM (~10× K_m). **b**–**v** Kinetics for different substate peptides (variable substrate), as indicated below each X-axis, with acetyl-CoA at a concentration of 500 µM (~10× K_m). K_m values for kinetics in subfigures **i, j, r** and **v** were below the smallest measured substrate concentrations and below the detection limit of the colorimetric assay. For these kinetics, the k_{cat} of each replicate was determined from average k_{obs} values for substrate concentrations above 100 µM. Dashed curves represent theoretical Michaelis-Menten curves for each replicate, assuming a K_m of 20 µM. In all four cases (subfigures **i, j, r** and **v**), the graphs clearly indicate that the real K_m value for these peptides is below 20 µM.

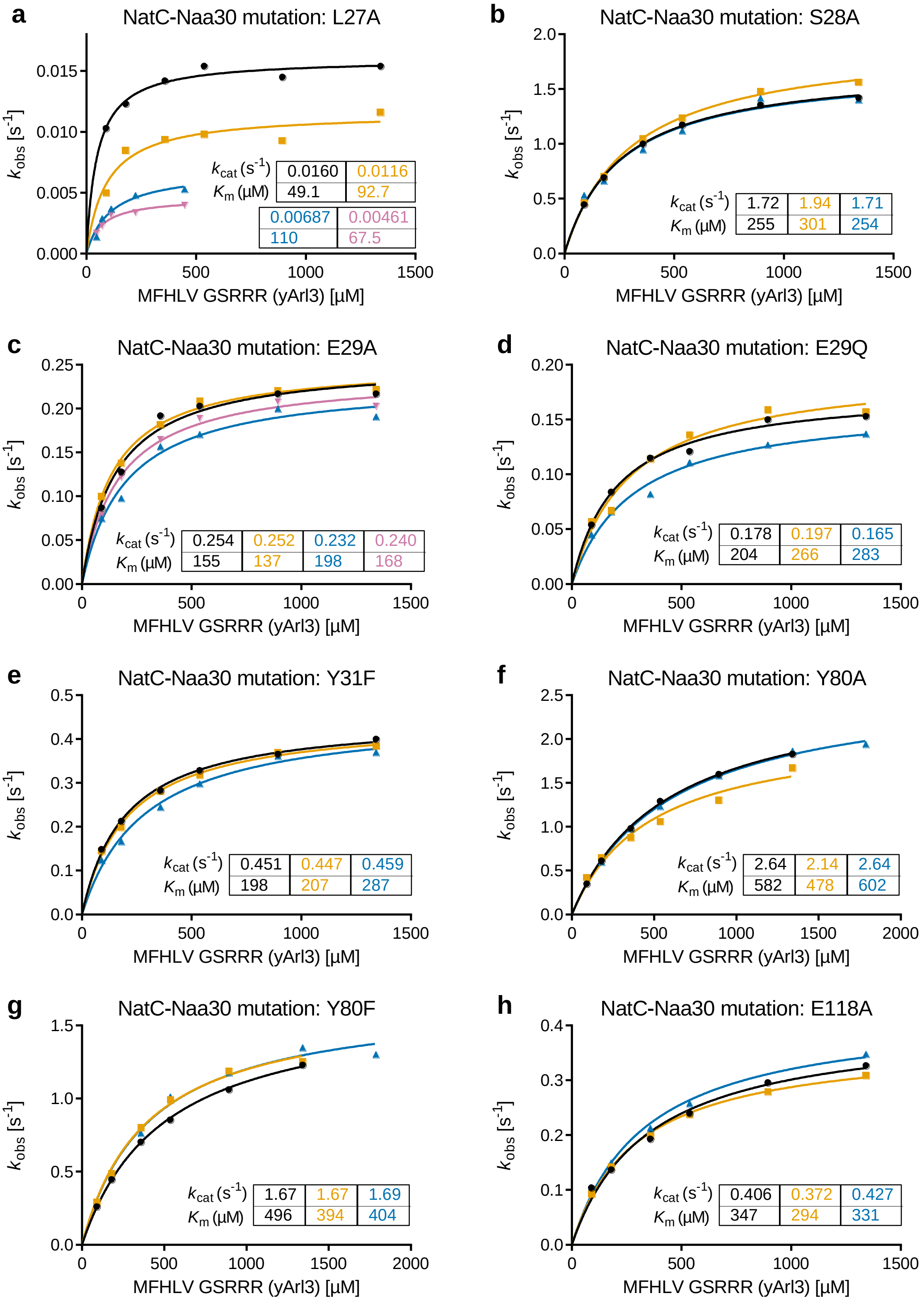
a
NatC-Naa30 mutation: L27A
k_{obs} [s^{-1}]
k_{cat} (s^{-1}) 0.0160 0.0116
K_m (μM) 49.1 92.7
0.00687 0.00461
110 67.5
MFHLV GSRRR (yArl3) [μM]
b
NatC-Naa30 mutation: S28A
k_{cat} (s^{-1}) 1.72 1.94 1.71
K_m (μM) 255 301 254
c
NatC-Naa30 mutation: E29A
k_{cat} (s^{-1}) 0.254 0.252 0.232 0.240
K_m (μM) 155 137 198 168
d
NatC-Naa30 mutation: E29Q
k_{cat} (s^{-1}) 0.178 0.197 0.165
K_m (μM) 204 266 283
e
NatC-Naa30 mutation: Y31F
k_{cat} (s^{-1}) 0.451 0.447 0.459
K_m (μM) 198 207 287
f
NatC-Naa30 mutation: Y80A
k_{cat} (s^{-1}) 2.64 2.14 2.64
K_m (μM) 582 478 602
g
NatC-Naa30 mutation: Y80F
k_{cat} (s^{-1}) 1.67 1.67 1.69
K_m (μM) 496 394 404
h
NatC-Naa30 mutation: E118A
k_{cat} (s^{-1}) 0.406 0.372 0.427
K_m (μM) 347 294 331

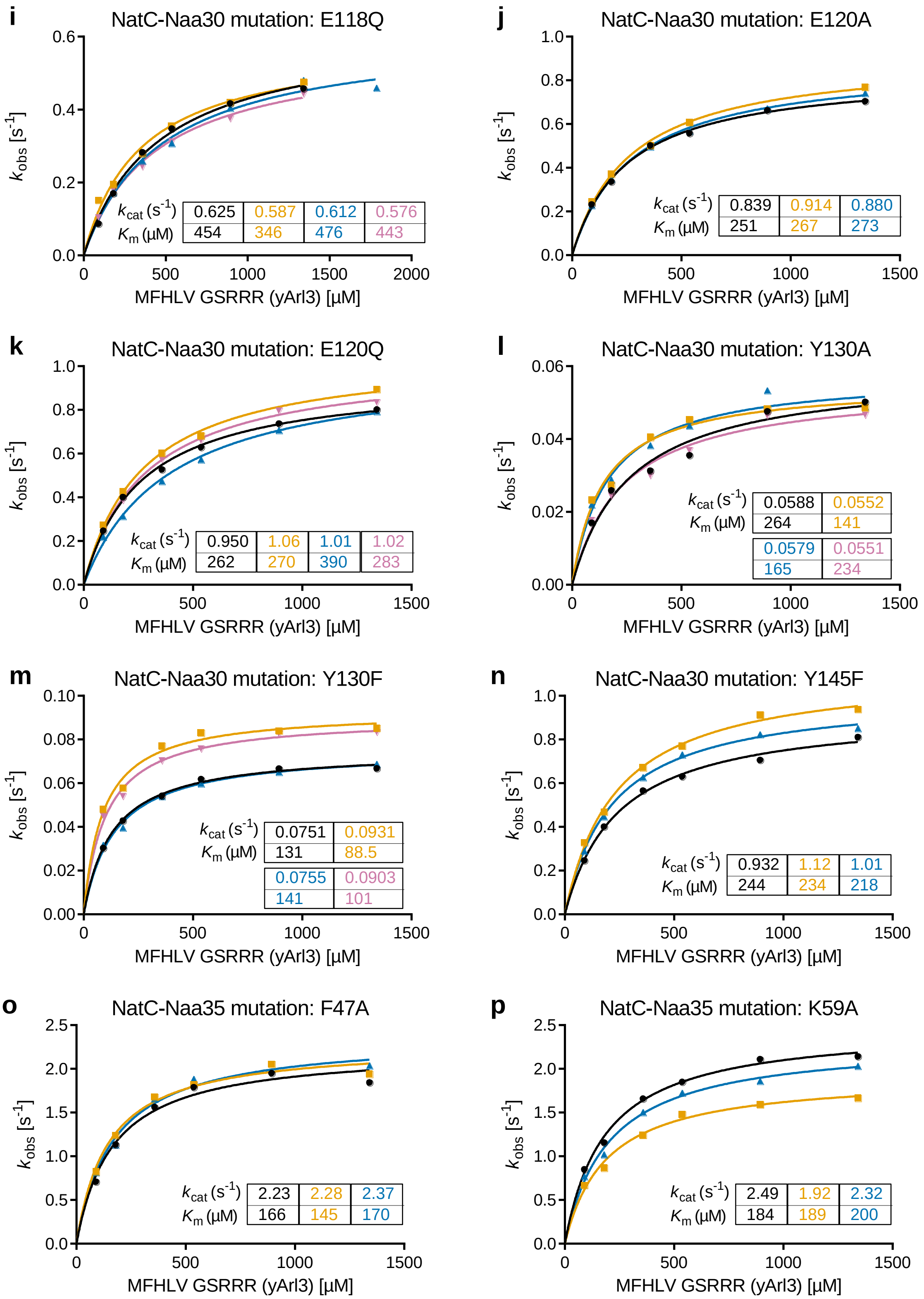
i
NatC-Naa30 mutation: E118Q
k_{obs} [s^{-1}]
MFHLV GSRRR (yArl3) [μM]
k_{cat} (s^{-1}) 0.625 0.587 0.612 0.576
K_m (μM) 454 346 476 443
j
NatC-Naa30 mutation: E120A
k_{cat} (s^{-1}) 0.839 0.914 0.880
K_m (μM) 251 267 273
k
NatC-Naa30 mutation: E120Q
k_{cat} (s^{-1}) 0.950 1.06 1.01 1.02
K_m (μM) 262 270 390 283
l
NatC-Naa30 mutation: Y130A
k_{cat} (s^{-1}) 0.0588 0.0552
K_m (μM) 264 141
0.0579 0.0551
165 234
m
NatC-Naa30 mutation: Y130F
k_{cat} (s^{-1}) 0.0751 0.0931
K_m (μM) 131 88.5
0.0755 0.0903
141 101
n
NatC-Naa30 mutation: Y145F
k_{cat} (s^{-1}) 0.932 1.12 1.01
K_m (μM) 244 234 218
o
NatC-Naa35 mutation: F47A
k_{cat} (s^{-1}) 2.23 2.28 2.37
K_m (μM) 166 145 170
p
NatC-Naa35 mutation: K59A
k_{cat} (s^{-1}) 2.49 1.92 2.32
K_m (μM) 184 189 200

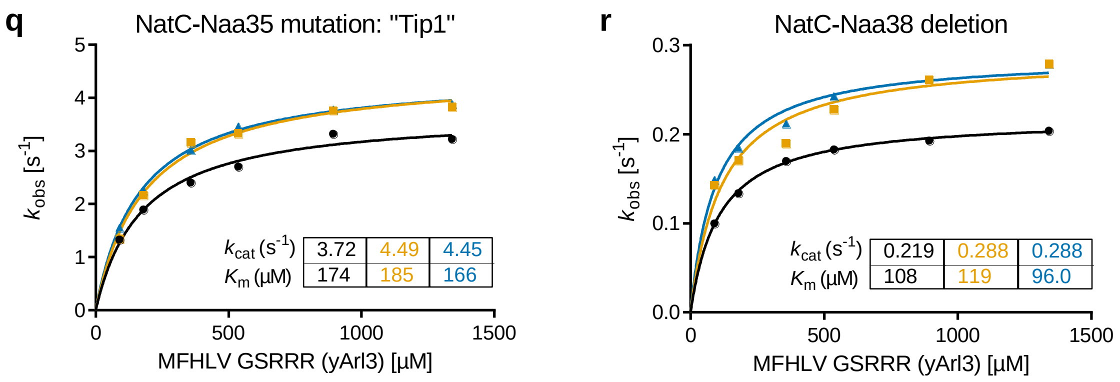

Supplementary Fig. 7 Michaelis-Menten kinetics for NatC mutants. All NatC mutants are based on the NatC WT (Naa30ΔC17, full-length Naa35, Naa38ΔC11) construct. Point mutations and the affected subunit are specified above each subfigure. All reactions were performed with the yArl3 peptide (MFHLVGSRRR) at a concentration of 1340 µM (~10× K_m). k_{obs} values represent initial reaction velocities (v_0) divided by the NatC concentration. Each graph (colored in black, orange, blue, purple and green) represents an independent experiment. Catalytic parameters (K_m, k_{cat}) for each replicate are shown below the plots in the corresponding color. The Tip1 mutant contains Naa35 point mutations: K500A, K501A, K503A and K504A.

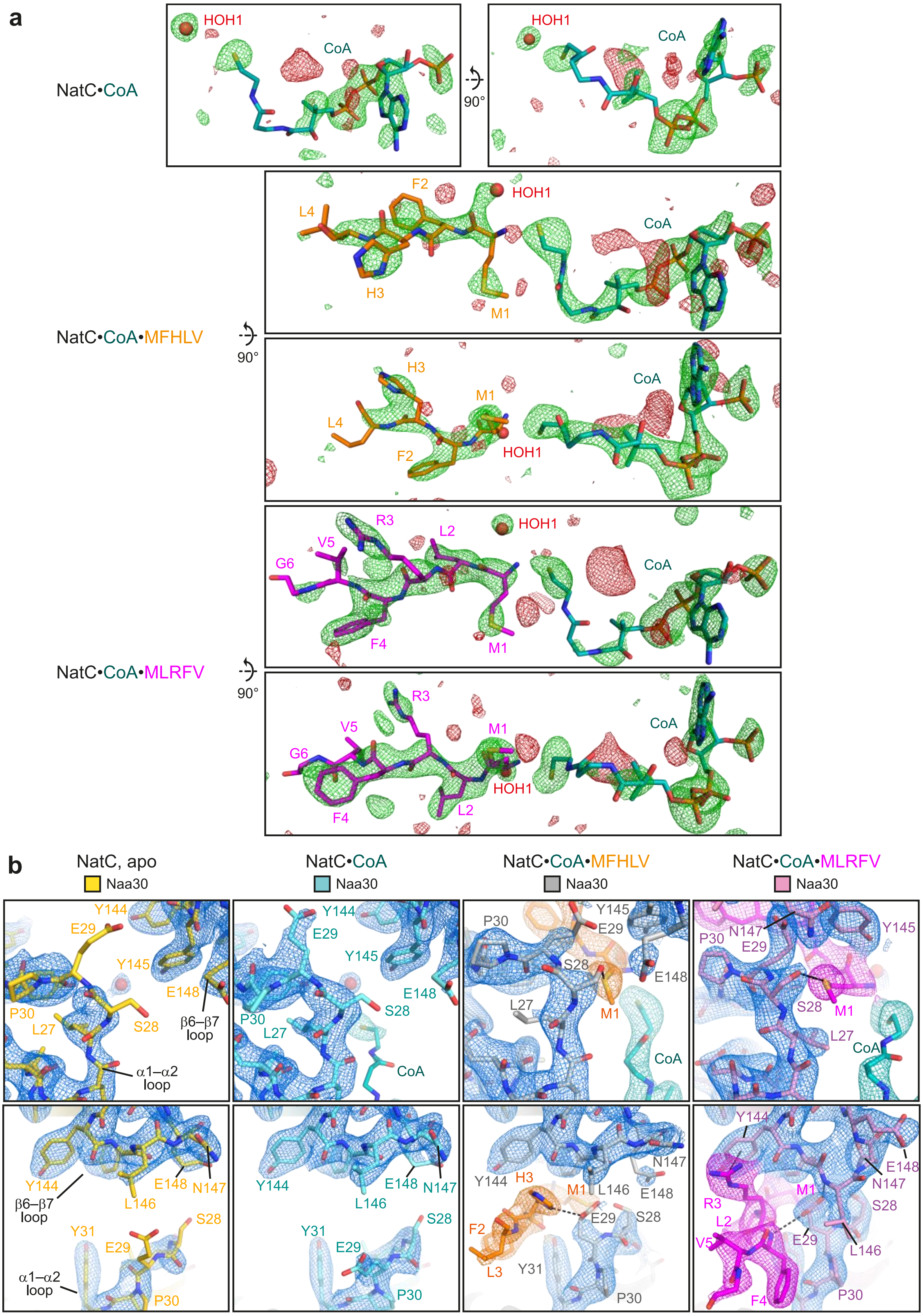

Supplementary Fig. 8 Electron density maps for the active site of NatC. a Simulated annealing OMIT maps (contoured to 3.0 σ) show positive (green) and negative (red) difference electron densities for CoA (teal), substrate peptides in the NatC•CoA•MFHLV (orange) and NatC•CoA•MLRFV (magenta) structures and a highly coordinated water molecule in the center of the active site (HOH1). **b** 2Fo-Fc maps (contoured at 1σ) for the α1–α2- and β1–β2 substrate-binding loops (blue) near the active site of the Naa30 subunit. 2Fo-Fc maps for CoA (teal), the yArl3 peptide (orange) and the Gag peptide (magenta) are shown in different colors for better differentiation.

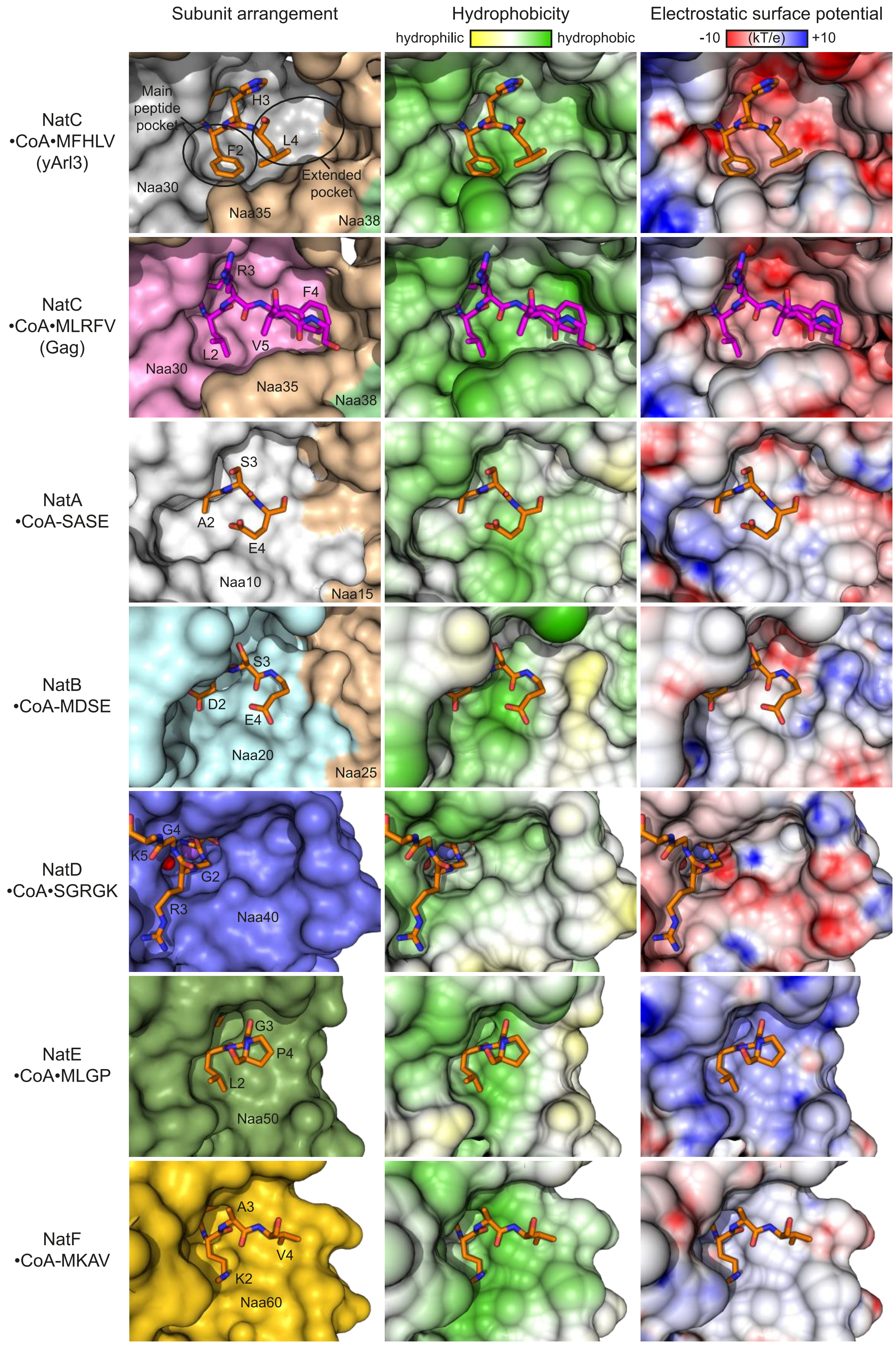

Supplementary Fig. 9 Comparisons of the peptide-binding surfaces in NAT orthologs. Substrate peptide recognition in the peptide-binding pocket of NatC•CoA•MFHLV, NatC•CoA•MLRFV, NatA•CoA-SASE (pdb 4KVM), NatB•CoA-MDSE (5K18), NatD•CoA•SGRGK (4U9W), NatE•CoA•MLGP (3TFY) and NatF•CoA-MKAV (5ICV). NAT complex subunits are colored individually to show their contribution to the peptide binding pocket (left). Additionally, NAT surfaces are colored according to hydrophobicity (middle) and electrostatic potential (right).

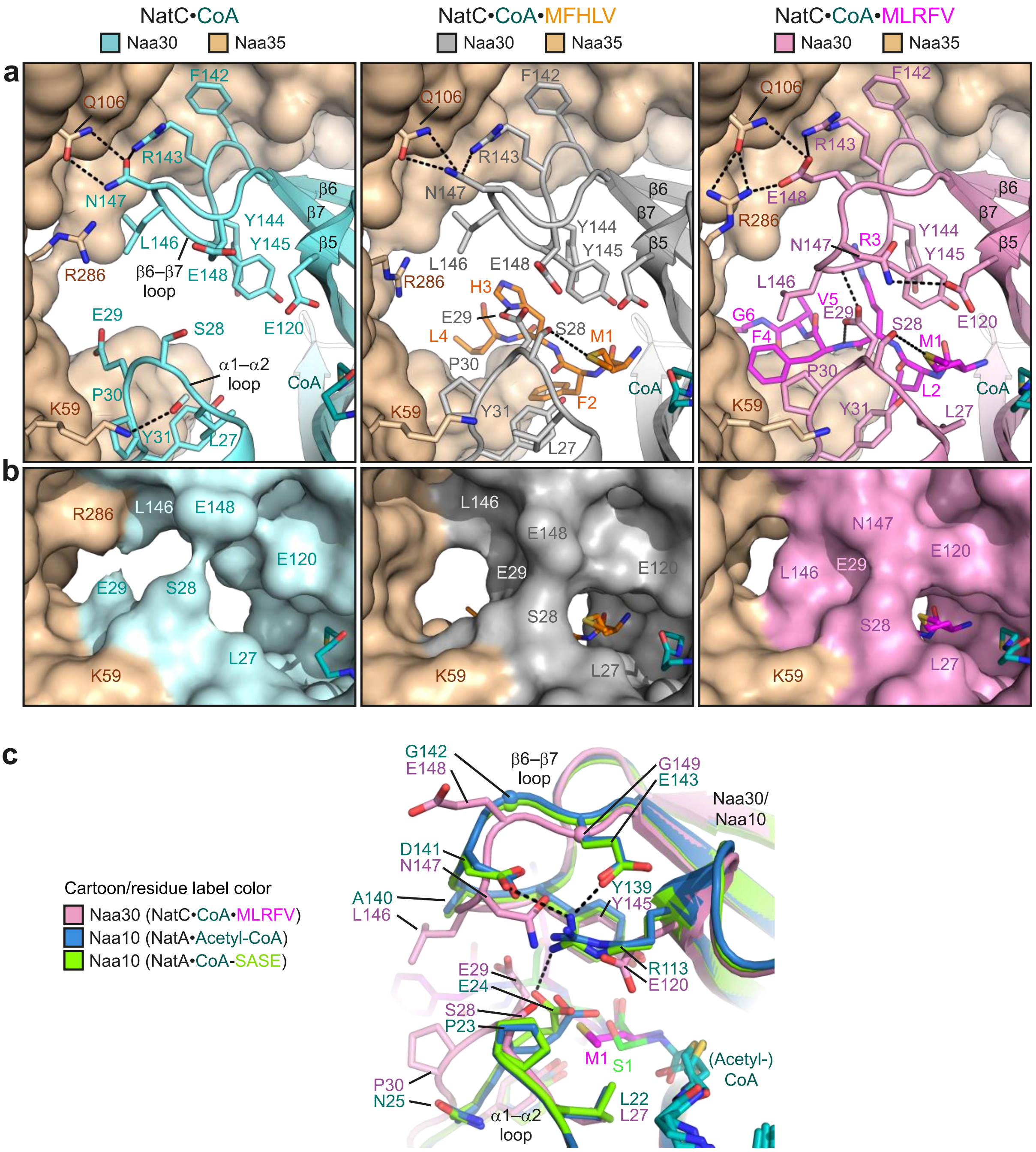

Supplementary Fig. 10 Peptide ligand-induced conformational changes. a Mixed surface (Naa35) and cartoon/stick (Naa30) representation, showing the structural rearrangements of the Naa30 α1–α 2 and β6–β7 loops upon ligand binding. **b** Surface projection of NatC, showing the effect of ligand binding on the shape and diameter of the central tunnel. **c** Superposition of Naa30 (NatC•CoA•MLRFV) with Naa10 of the *Schizosaccharomyces pombe* NatA complex in the presence of Acetyl-CoA (pdb 4KVO) or a bisubstrate conjugate CoA-SASE (4KVM).

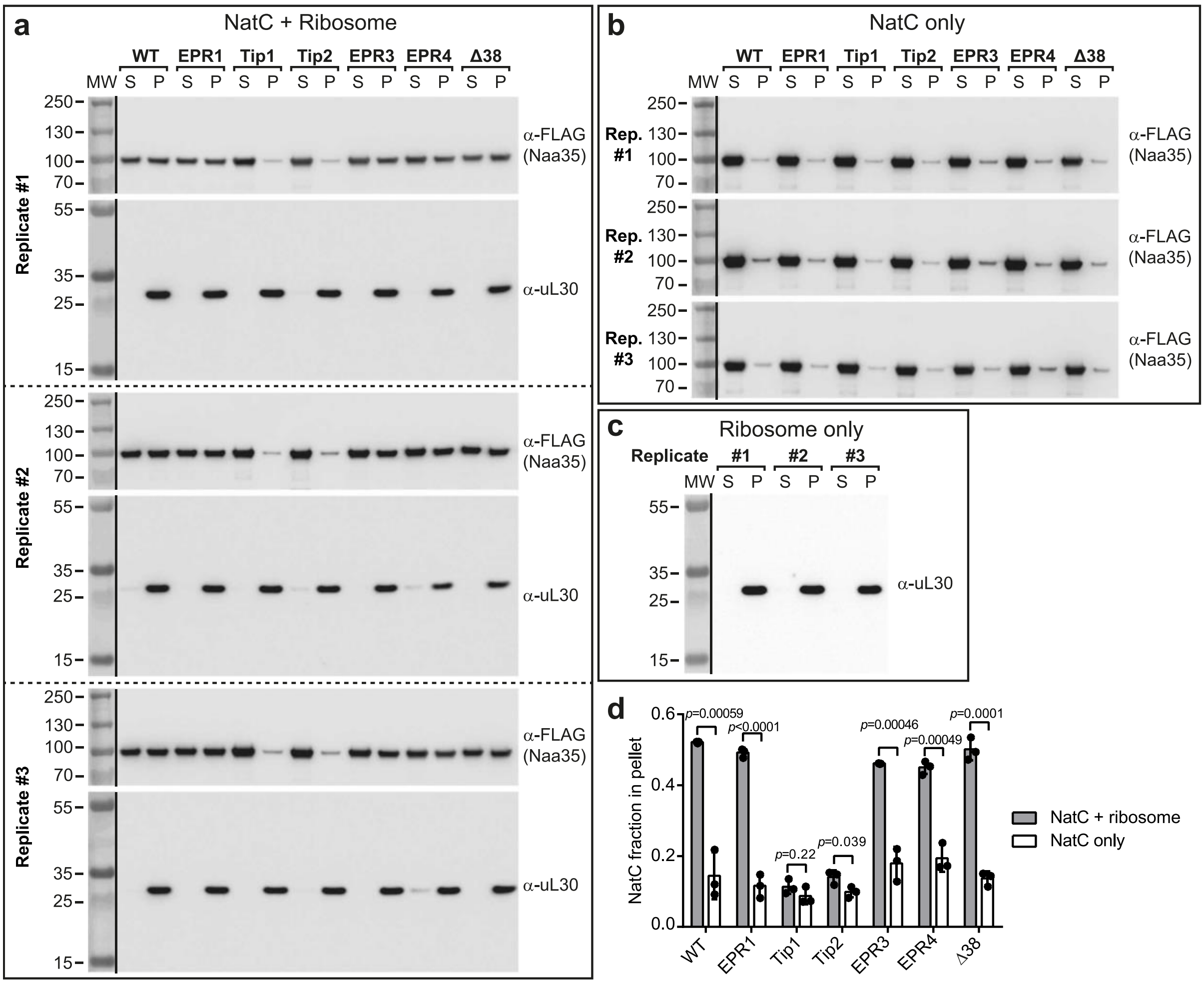

Supplementary Fig. 11 Replicates of the NatC/ribosome co-sedimentation assay. a–**c** Western blot of three individual replicates of the NatC/ribosome co-sedimentation assay (replicate #1 of Fig.11a,b is additionally shown in Fig. 5b). NatC constructs in the supernatant (S) and pellet (P) fractions were immunodetected via a FLAG tag at the Naa35 N-terminus; yeast ribosomes via the ribosomal protein uL30. EPR, electropositive region. **b** NatC sedimentation control. **c** Ribosome sedimentation control. All images in a–c show the molecular weight marker (MW) in lane 1 as an incident white light image, while all remaining lanes are chemoluminescence images from the same blot. **d** Quantification (chemoluminescence band intensities) of the NatC fraction in the pellet (P/(S+P)) of subfigures **a** and **b**. All data represent mean ± SD (*n*=3 independent experiments). NatC/ribosome co-sedimentation experiments were compared to the NatC sedimentation control using unpaired two-tailed t-tests with the Holm-Sidak correction. Uncropped images for panels a–c are provided in the Source Data file.

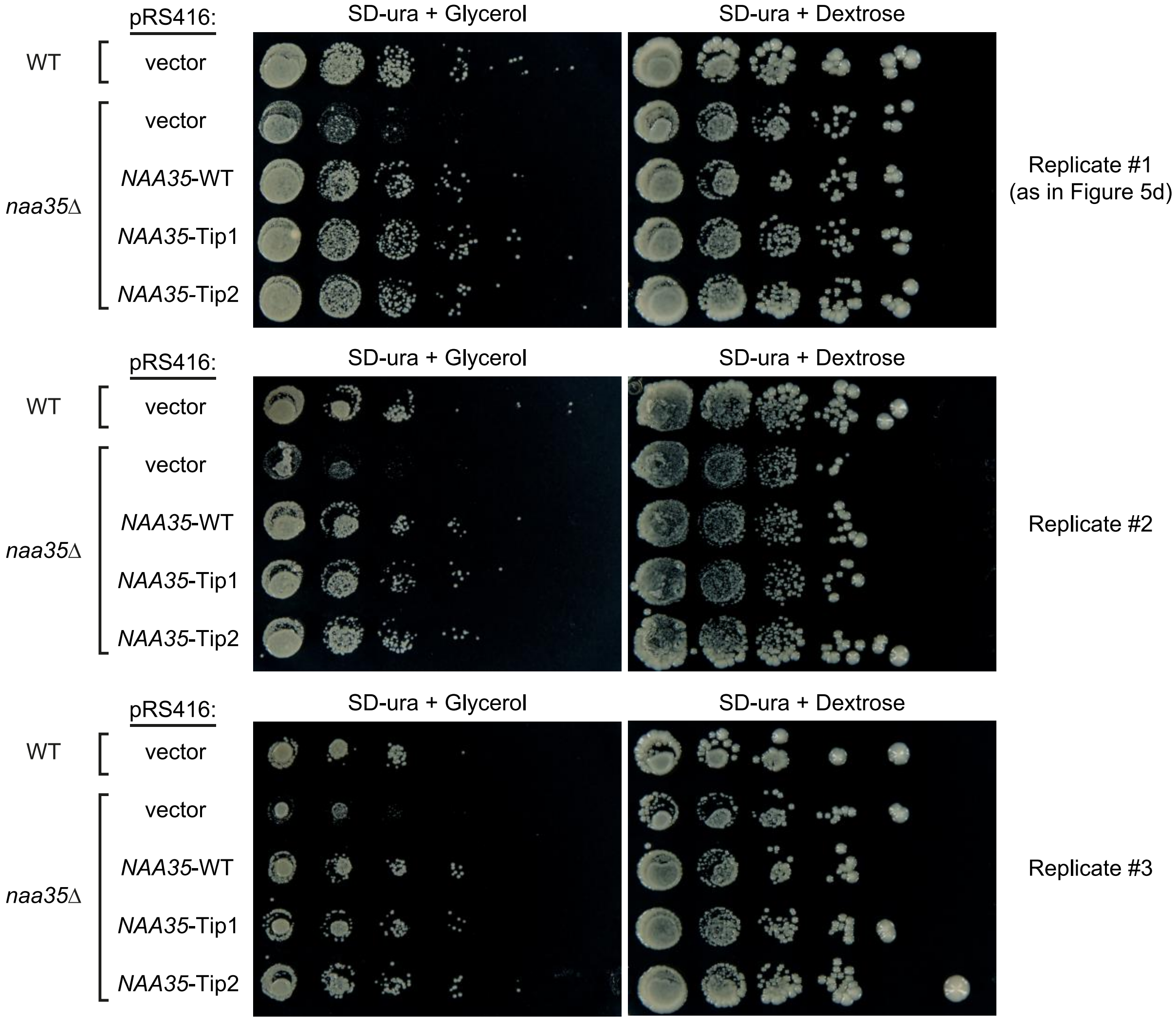

Supplementary Fig. 12 Replicates of the yeast dilution spot assay. Three individual replicates of the yeast dilution spot assay obtained for individually transformed yeast cells (replicate #1 is additionally shown in Fig. 5d). SD-ura, synthetic defined dropout medium without uracil. Serial tenfold dilutions of *S. cerevisiae* WT (BY4741) and *naa35*Δ (Y00294) strains, transformed with a pRS416 yeast centromere vector, carrying no insert (vector), *NAA35*-WT or the *Naa35-Tip1* (K500A, K501A, K503A, K504A) or *Naa35-Tip2* mutant (K511A, R515A, R519A). Cells were grown at 37 °C for 5 days on SD-ura agar plates, supplemented with 3% glycerol or 2% dextrose, respectively.

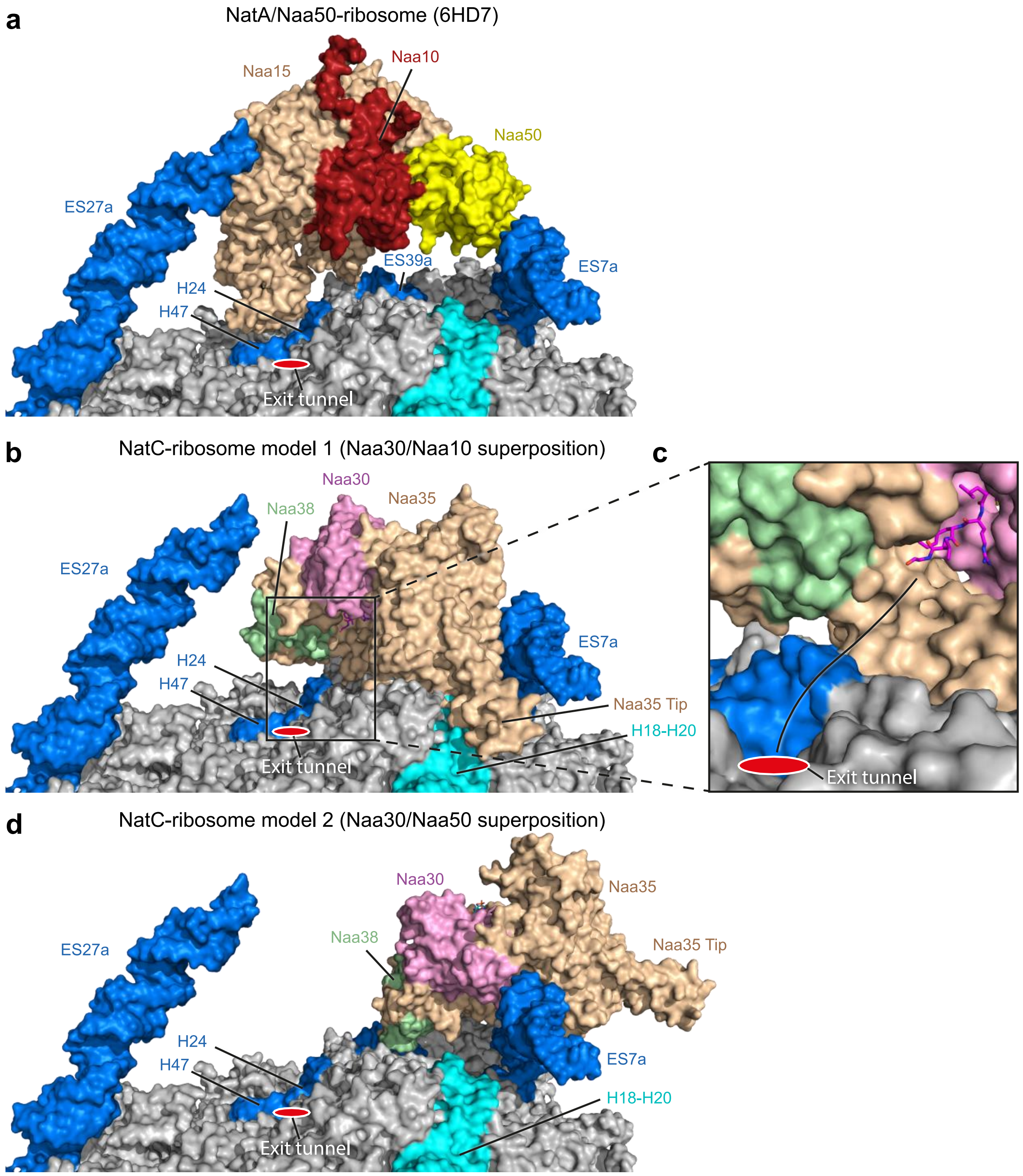

Supplementary Fig. 13 Models of NatC on the ribosomal surface. a Molecular model of the NatA/Naa50-ribosome complex (6HD7). NatA/Naa50 interacts with ribosomal RNA expansion segments (ES) and RNA helices (H), both colored in blue, near the ribosomal exit tunnel. The catalytic subunit Naa30 of the NatC•CoA•MLRFV structure was used for a superposition with Naa10 (RMSD = 1.10 Å over Cα 127 atoms) or Naa50 (RMSD = 2.70 Å over Cα 124 atoms) of the NatA/Naa50-ribosome complex: **b** Model 1 of the NatC-ribosome complex, generated by a Naa30/Naa10 superposition. In this model, the auxiliary subunit Naa35 is in close contact with the surface of the ribosome. Moreover, the Naa35 tip region would contact ribosomal helices H18–H20 (cyan). **c**, Magnified view of model 1, showing the close proximity between peptide-binding site and the ribosomal exit tunnel. This model would thus be consistent with a conserved arrangement of the acetylation centers in NatA and NatC relative to nascent polypeptide chain emerging from the ribosome. **d** Model 2 of the NatC-ribosome complex, generated by a superposition of Naa30 with Naa50. Hardly any interaction between NatC and the ribosome was observed and the Naa35 tip is positioned far away from the ribosome surface, arguing against model 2 representing a native conformation.

Supplementary References

1. Gibson DG, Young L, Chuang RY, Venter JC, Hutchison CA, 3rd, Smith HO. Enzymatic assembly of DNA molecules up to several hundred kilobases. *Nat Methods* **6**, 343-345 (2009).
