# Supplementary material for: Divergent architecture of the heterotrimeric NatC complex explains N-terminal acetylation of cognate substrates
Source: Nat Commun. 2020 Nov 2;11:5506. doi: 10.1038/s41467-020-19321-8 (PMC7608589; doi:10.1038/s41467-020-19321-8)
Supplement: Supplementary file 3 — Reporting Summary [file 41467_2020_19321_MOESM3_ESM.pdf]

nature research

Corresponding author(s): Oliver Daumke

Last updated by author(s): Sep 28, 2020

Reporting Summary

Nature Research wishes to improve the reproducibility of the work that we publish. This form provides structure for consistency and transparency in reporting. For further information on Nature Research policies, see our Editorial Policies and the Editorial Policy Checklist.

Statistics

For all statistical analyses, confirm that the following items are present in the figure legend, table legend, main text, or Methods section.

n/a	Confirmed	
☐	☒	The exact sample size (n) for each experimental group/condition, given as a discrete number and unit of measurement
☐	☒	A statement on whether measurements were taken from distinct samples or whether the same sample was measured repeatedly
☐	☒	The statistical test(s) used AND whether they are one- or two-sided<br>*Only common tests should be described solely by name; describe more complex techniques in the Methods section.*
☒	☐	A description of all covariates tested
☐	☒	A description of any assumptions or corrections, such as tests of normality and adjustment for multiple comparisons
☐	☒	A full description of the statistical parameters including central tendency (e.g. means) or other basic estimates (e.g. regression coefficient) AND variation (e.g. standard deviation) or associated estimates of uncertainty (e.g. confidence intervals)
☐	☒	For null hypothesis testing, the test statistic (e.g. F, t, r) with confidence intervals, effect sizes, degrees of freedom and P value noted<br>*Give P values as exact values whenever suitable.*
☒	☐	For Bayesian analysis, information on the choice of priors and Markov chain Monte Carlo settings
☒	☐	For hierarchical and complex designs, identification of the appropriate level for tests and full reporting of outcomes
☒	☐	Estimates of effect sizes (e.g. Cohen's d, Pearson's r), indicating how they were calculated

Our web collection on statistics for biologists contains articles on many of the points above.

Software and code

Policy information about availability of computer code

Data collection	XDS (Version: Mar 15, 2019)
Data analysis	XDS (Version: Mar15, 2019), Phenix-1.11.1-2575, Coot 0.8.9.1, CCP4 interface 7.0.017, HKL2Map 0.4.e interface for ShelxC (2016/1), ShelxD (2013/2), ShelxE (2018/2), Buccaneer v1.5, PDBe PISA v1.52, DALI v.4.0 web server, PyMOL 2.3.1., APBS PyMOL plugin (PyMOL incentive version 2.3.1) , VASCo PyMOL plugin v0.77beta, ESPript 3.0, Geneious 11.0, GraphPad Prism 6.01, Microsoft Excel 365, ImageJ 1.52a

For manuscripts utilizing custom algorithms or software that are central to the research but not yet described in published literature, software must be made available to editors and reviewers. We strongly encourage code deposition in a community repository (e.g. GitHub). See the Nature Research guidelines for submitting code & software for further information.

Data

Policy information about availability of data

All manuscripts must include a data availability statement. This statement should provide the following information, where applicable:

- Accession codes, unique identifiers, or web links for publicly available datasets
- A list of figures that have associated raw data
- A description of any restrictions on data availability

The atomic coordinates of NatC have been deposited in the Protein Data Bank with accession numbers 6YGA (NatC, apo), 6YGB (NatC•CoA), 6YGC (NatC•CoA•MFHLV) and 6YGD (NatC•CoA•MLRFV). The source data underlying Table 2, Figs. 3a, 4b, 5c, and Supplementary Figs. 6a–v , 7a–r and 11a–d are provided in a separate Source Data file.

Field-specific reporting

Please select the one below that is the best fit for your research. If you are not sure, read the appropriate sections before making your selection.

☒ Life sciences ☐ Behavioural & social sciences ☐ Ecological, evolutionary & environmental sciences

For a reference copy of the document with all sections, see nature.com/documents/nr-reporting-summary-flat.pdf

Life sciences study design

All studies must disclose on these points even when the disclosure is negative.

Sample size	No statistical methods were used to predetermine sample size. X-ray diffraction data were collected until completeness of the data set. Acetyltransferase assays and ribosome-spin down assays were repeated with n of at least, 3 to allow calculation of SD. Replicates represent individual experiments using the same biological sample. The sample size was adequate, since the differences between experimental groups was reproducible.
Data exclusions	No data were excluded.
Replication	The experimental findings were reproduced in multiple independent experiments. The number of independent experiments and biological replicates in each data panel is indicated in the figure legends.
Randomization	Randomization was not formerly performed in this study as it did not involve animals and/or human research participants. Covariates, like buffer compositions, assay temperature and protein preparations were controlled, as all experiments were performed under identical conditions for all experimental groups for both the acetlytransferase assays, as well as the ribosome spin-down assay.
Blinding	Blinding is not relevant for protein structure determination since the results are not subjective. Investigators were not blinded during the functional assays, since kinetic Michaelis-Menten parameters are not subjective.

Reporting for specific materials, systems and methods

We require information from authors about some types of materials, experimental systems and methods used in many studies. Here, indicate whether each material, system or method listed is relevant to your study. If you are not sure if a list item applies to your research, read the appropriate section before selecting a response.

Materials & experimental systems

n/a	Involved in the study	
☐	☒	Antibodies
☐	☒	Eukaryotic cell lines
☒	☐	Palaeontology and archaeology
☒	☐	Animals and other organisms
☒	☐	Human research participants
☒	☐	Clinical data
☒	☐	Dual use research of concern

Methods

n/a	Involved in the study	
☒	☐	ChIP-seq
☒	☐	Flow cytometry
☒	☐	MRI-based neuroimaging

Antibodies

Antibodies used	Primary antibodies: mouse α-FLAG M2 (Sigma-Aldrich, F3165) and rabbit α-uL30/α-RPL7, (Abcam, ab72550).<br>Secondary antibodies: goat IgG α-Mouse IgG (H+L)-HRPO, MinX none (Dianova GmbH, 115-035-003) and goat IgG α-Rabbit IgG (H+L)-HRPO, MinX none (Dianova GmbH, 111-035-003)
Validation	The α-FLAG M2 antibody (Sigma-Aldrich, F3165) was already tested in many peer-reviewed studies, for example Han et al. 2019 (https://www.nature.com/articles/s41467-019-11618-7). Additionally, the antibody was validated in this study using recombinantly purified, N-terminally FLAG-tagged S.cerevisiae NatC protein. A single chemoluminescence band was present at the expected molecular weight (85.5 kDa) for the recombinant fusion protein. No additional band(s) were visible on the western blot.<br><br>The α-uL30/α-RPL7 antibody (Abcam, ab72550) was validated using purified S. cerevisiae ribosomes. A single chemoluminescence band was present at the expected molecular weight (27.6 kDa) for yeast RPL7A (Uniprot ID: P05737). No additional band(s) were visible on the western blot.

Eukaryotic cell lines

Policy information about cell lines

Cell line source(s)	S. cerevisiae wild type (BY4741) and Naa35-deletion (Y00294) strains were obtained from Euroscarf.
Authentication	We confirmed the Naa35 gene knockout by PCR.
Mycoplasma contamination	n/a.
Commonly misidentified lines (See ICLAC register)	n/a.
